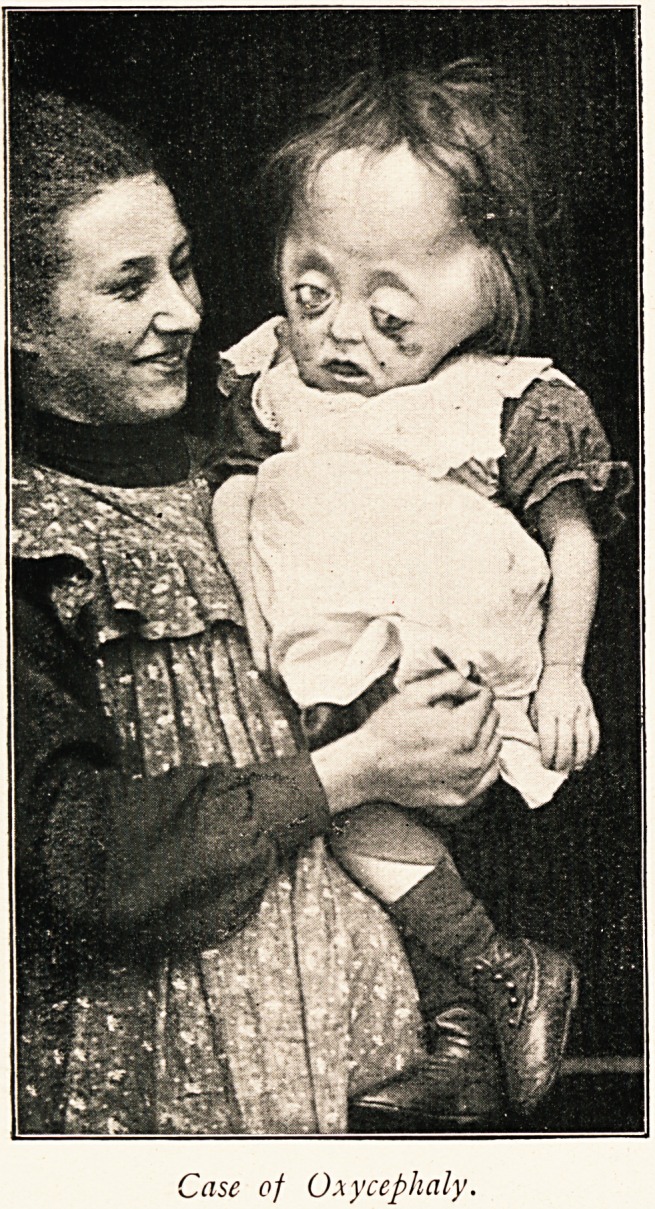# Notes on a Case of Oxycephaly

**Published:** 1910-06

**Authors:** G. Hely-Hutchinson Almond


### NOTES ON A CASE OF OXYCEPHALY.

BY

G. HELY-HUTCHINSON ALMOND, M.B., M.A.Oxon.

J. D., a male child, was born on October 22nd, 1908. His mother was then 34, and had previously given birth to seven normal children, four girls and three boys, the youngest of whom was a girl, 1 year and 7 months old. All the children are now alive and well. At the time of writing—May, 1910—another girl has just been born, and she appears to be quite normal.

There is no history of syphilis in either father or mother. The former, at the time of birth, was suffering from a sinus in the lower jaw, the result of necrosis ; this has now quite healed. The mother has never suffered from vaginal discharge, and has had no miscarriages. She has breast-fed all her babies for the normal period.

Four months before the birth of the patient the mother received a severe fright on seeing a burning child, and to this fact she attributed the deformity. The confinement was attended by a midwife. Though the labour only lasted seven hours, the head was rather slow in being born. The baby was not weighed, permission being withheld by the parents, but it was estimated to be about 9 lb. The skin was natural in colour and texture. It was brought up to me to see on account of the shape of its head.

The forehead had a characteristic dome-shaped appearance, rising abruptly from the orbital ridges. The anterior fontanelle was open, the posterior much so. There were large temporal bulgings on each side, and the antero-lateral fontanelles were widely patent. Proptosis was marked, and there was some ectropion. When five weeks old the occipito-mental circumference of the head was 17 inches, and the occipito-frontal 15. If callipers had been used to measure, the occipito-frontal diameter would have been comparatively very much less, as the tape, in taking the circumference, had to pass over the temporal bulgings.

The lateral aspects of the forehead are now very much bossed, and the frontal suture runs up between them as a shallow depression. The anterior fontanelle is just open. The posterior fontanelle is closed, but close to it the left lambdoid suture is widely open, and through it proceeds an encephalocele about 1½ inches in diameter. The temporal bulging on the right side is now closed over with bone, but on the left there is the considerable diamond-shaped opening of the fontanelle. Both postero-lateral fontanelles only just admit the pulp of the finger. The following is a table of measurements taken at various times :—

| | *Nov.*, 1908. | *May*, 1909. | *May*, 1910. |
|---|---|---|---|
| Occipito-frontal .. .. .. .. | 15 | 17½ | 19 |
| Occipito-mental .. .. .. .. | 17 | 18 | 24½ [1] |
| Level of eyes .. .. .. .. | — | 19 | 22 |
| From ext. aud. meatus of one side over vertex to other side .. .. .. .. | — | 15½ | 16 |

The eye sockets are very shallow. A considerable portion of the roof of the orbital cavity, which is much more vertical than normal, can be easily palpated without displacement of the eyeball. Exophthalmos has been marked since birth. Dislocation of either eyeball occurs occasionally when the child cries. During

[1] Partly accounted for by enlargement of cephalocele.

the first year no squint was observed, but for the last six or seven months the child has been fixing with his right eye, whilst the axis of the left eye points a little downwards and outwards, and this tendency is increasing. Vision is good, and the child can easily distinguish people in the gardens at the opposite side of the road, *i.e.* at a distance of about forty feet. Nystagmus has never been found. The eyelids at times cover the eyes when the child is asleep, but at other times closure is imperfect, especially when the child has had an attack of chemosis, a not infrequent event. These bouts are always associated with conjunctivitis, and once the chemosis in the left eye was so severe that in order to relieve tension the conjunctiva had to be punctured. The discs are natural. The bridge of the nose is depressed.

The palate is very highly arched, so much so that it is impossible for the index finger to reach the apex of the arch. The upper jaw is rather contracted, the central portion containing the incisors being somewhat bowed out as a buttress. Two teeth were cut before three months, and the remainder of the sixteen before one year. The upper incisors are rather crowded together.

The chest is fairly well developed. The heart is natural, and the lungs are sound. The abdomen is natural, the limbs are thin and flabby. The child is unable to walk, and has only recently been able to stand with support.

In December, 1909, the baby had measles, followed by a sharp attack of bronchitis. In March, 1910, he woke up one afternoon from sleep, began to sweat, vomited and followed this up with tonic and clonic convulsions. The fit was followed by another bout of bronchitis.

The accompanying photograph, kindly taken for me by Mr. H. K. Pvyke, shows the patient taken with his eldest sister (age 15). It is interesting to compare the measurements of their heads.

| | | | | | |
|---|---|---|---|---|---|
| Patient | .. | O.M. | $24\frac{1}{2}$; | O.F. | 19. |
| Sister | .. | O.M. | $25\frac{1}{4}$; | O.F. | $21\frac{1}{4}$. |

The ætiology of the condition is uncertain. I do not attach any importance to the mother's fright.

*Case of Oxycephaly.*

Carpenter[1] shows that for congenital heart cases the theory of maternal impressions does not hold water, adducing as proof that the fœtal heart is in miniature perfectly formed seven weeks after conception. Unless of course, as is quite possible, the cause of the condition had been at work before the end of the fifth month, his remarks as regards the present case can only be of indirect value.

The malformation has been ascribed to intra-uterine meningitis, which is supposed to cause early union of the sutures. There is no evidence in the history of the mother to prove this, but it is, at the same time, impossible to deny its existence.

There is, however, in this and in other cases evidence of the early union of some at least of the sutures, and the well-marked frontal groove in the present instance bears this out. The early appearance of the teeth I consider brings up the possibility of some hyperactivity of the osteoplastic tissues.

The early union of the sutures, especially of the coronal and sagittal, leads in some cases to a microcephalic oxycephaly, such as was described in one of Mr. Power's cases,[2] or depicted in one of Dr. Morley Fletcher's cases.[3]

In other cases, such as in my own, some of the fontanelles and sutures—the points of weakest resistance—have apparently yielded to the increased pressure put upon them, and this has led to the formation of encephaloceles, and at the same time has possibly indirectly paved the way for an accompanying hydrocephaly.

The malformation of the orbit is generally supposed to be due to synostoses affecting the base of the skull.

Mr. Beaumont has seen the child with me several times, and has already alluded to it in the *Transactions of the Ophthalmological Society*.[4] To him I am indebted for several valuable suggestions.

[1] *Proc. Roy. Soc. Med.*, Child. Sect., 1909, ii, 277.
[2] *Tr. Ophth. Soc. Lond.*, 1894, xiv, 212.
[3] *Proc. Roy. Soc. Med.*, Clinical Sect., 1909, ii, 115.
[4] *Tr. Ophth. Soc. Lond.*, 1910, xxx, 47.

## Figures and Tables

**Figure f1:**